# Supplementary material for: Splice-Junction-Based Mapping of Alternative Isoforms in the Human Proteome
Source: Cell Rep. Author manuscript; Available in PMC 2020 Jan 15. (PMC6961840; doi:10.1016/j.celrep.2019.11.026)
Supplement: 2 [file NIHMS1546469-supplement-2.zip › DF1/PXD006675/Aorta/Aorta_6_SRRM1_KVELSESEDK.pdf]

A

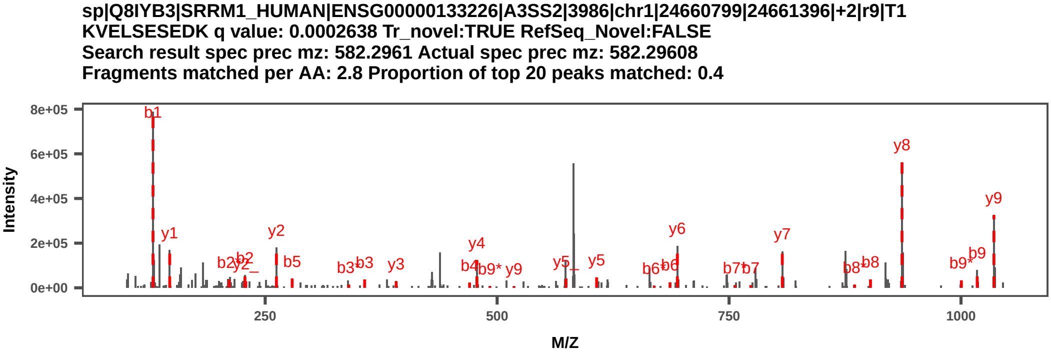
sp|Q8IYB3|SRRM1_HUMAN|ENSG00000133226|A3SS2|3986|chr1|24660799|24661396|+2|r9|T1
KVELSESEDK q value: 0.0002638 Tr_novel:TRUE RefSeq_Novel:FALSE
Search result spec prec mz: 582.2961 Actual spec prec mz: 582.29608
Fragments matched per AA: 2.8 Proportion of top 20 peaks matched: 0.4
Intensity
M/Z
8e+05
6e+05
4e+05
2e+05
0e+00
250
500
750
1000
b1
y1
y2
y8
y9
y6
y7
y4
y5
y3
b5
b3
b8
b9

B

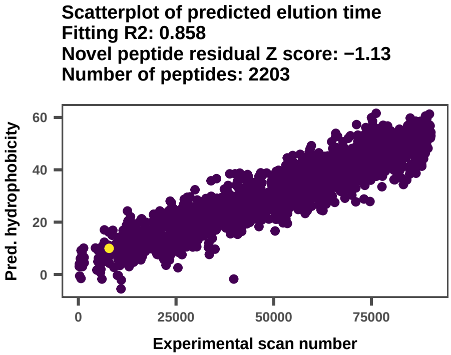
Scatterplot of predicted elution time
Fitting R2: 0.858
Novel peptide residual Z score: −1.13
Number of peptides: 2203
Pred. hydrophobicity
Experimental scan number
60
40
20
0
25000
50000
75000

C

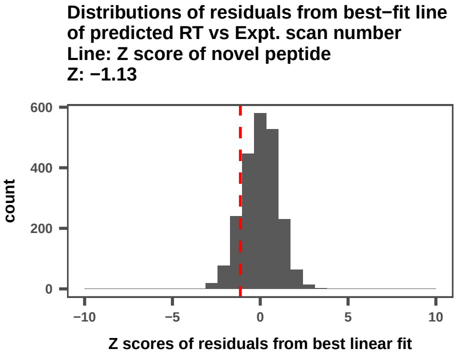
Distributions of residuals from best−fit line
of predicted RT vs Expt. scan number
Line: Z score of novel peptide
Z: −1.13
count
Z scores of residuals from best linear fit
600
400
200
0
−10
−5
5
10
